# Experimental Study on Anti-Wrinkling Performance of TA1 Titanium Thin Sheet Assisted by Ultrasonic Vibration

**DOI:** 10.3390/ma18071439

**Published:** 2025-03-24

**Authors:** Jiayi Ma, Yucheng Wang, Chunju Wang, Haidong He, Feng Chen, Lining Sun

**Affiliations:** School of Mechanical and Electrical Engineering, Robotics and Microsystems Center, Soochow University, Suzhou 215131, China; 20244229012@stu.suda.edu.cn (J.M.); 20225229103@stu.suda.edu.cn (Y.W.); chenfeng508@suda.edu.cn (F.C.); lnsun@hit.edu.cn (L.S.)

**Keywords:** ultrasonic-vibration energy field, TA1 titanium sheet, plate diagonal tensile testing, anti-wrinkling performance

## Abstract

TA1 titanium bipolar plates for hydrogen fuel cells are prone to plastic instability phenomena such as wrinkling during the stamping process, which adversely affects the forming quality. This study applies an ultrasonic-vibration energy field, aligned with the direction of stretching, in a plate diagonal tensile testing scenario based on the Blaha effect. The impact of varying thicknesses and vibration amplitudes on the anti-wrinkling performance of TA1 titanium sheets is investigated. Through a combined analysis of load–displacement curves and wrinkle height measurements using a super-depth-of-field microscope, by examining the forming load, the onset of wrinkling, and the wrinkle height at buckling locations, this study explores the deformation behavior of the thin sheet and the wrinkle suppression mechanism under the coupled effects of the ultrasonic-vibration field and scale. The results show that as the thickness decreases, the anti-wrinkling ability of the TA1 titanium sheet diminishes. The ultrasonic-vibration energy field reduces the yield stress and flow stress of the material, promoting wrinkling during the elastic deformation stage. Moreover, the 0.075 mm thick TA1 titanium sheet experiences local secondary wrinkling during the plastic deformation stage. Additionally, the ultrasonic-vibration energy field effectively reduces the forming load of the sheet and suppresses wrinkling within a certain range of amplitudes. These findings provide experimental evidence for the ultrasonic-vibration-assisted stamping process of titanium bipolar plates.

## 1. Introduction

Hydrogen fuel cells directly convert chemical energy into electrical energy. Recognized globally as a zero-emission, efficient, and renewable energy source, hydrogen fuel cells play a crucial role in the transition to sustainable energy [1]. Bipolar plates are essential components of fuel cells, responsible for evenly distributing reaction gases, collecting current, providing cooling, and offering mechanical support. They account for more than 60% of the weight and over 30% of the cost of the cell [2]. Currently, the manufacturing of metallic bipolar plates primarily relies on stamping processes [3], with TA1 titanium commonly being used due to its lightweight properties and enhanced corrosion resistance.

However, during the stamping process, which is characterized by tensile strain and the relatively low elongation of titanium sheets, phenomena such as wrinkling and cracking often occur, leading to plastic instability. These issues degrade forming quality, potentially damage molds, and increase production costs [4]. Wrinkling, a typical manifestation of compressive instability in thin-sheet stamping, occurs when the transverse compressive stress exceeds the critical buckling stress, causing the material to transition from uniform in-plane deformation to out-of-plane buckling [5]. In the 1980s, Yoshida [6] introduced the Yoshida Buckling Test (YBT) to study the wrinkling behavior of sheets under non-uniform axial loads. The simplicity of the YBT model, the ease of data extraction, and its ability to assess the anti-wrinkling capacity of materials have made it a standard method for wrinkling research. Numerous studies have been based on the YBT to explore the influence of material properties [7], boundary conditions [8,9,10], and geometric shapes [11,12,13] on the wrinkling resistance of thin sheets.

Despite significant progress in the study of wrinkling instability in thin plates, and notable advancements in enhancing anti-wrinkling performance and wrinkle prediction, the increasing demands for reduced volume, weight, and cost in fuel cells have led to a continuous decrease in the thickness of metallic bipolar plates. Consequently, traditional stamping processes face mounting challenges, primarily manifested in increased wall-thinning ratios, greater difficulty in controlling microchannel dimensional accuracy, and reduced flatness at rib crests. These issues result in a non-ideal contact surface between the metallic bipolar plate and the proton exchange membrane (PEM). To ensure adequate contact, a preload must be applied, leading to uneven stress distribution across the contact region, which significantly affects key parameters such as contact resistance and PEM porosity. Ultimately, these factors degrade the fuel cell’s output power, performance uniformity, and service life. To mitigate wrinkling and warping issues associated with rigid die stamping, various anti-wrinkling strategies have been proposed, including hydraulic forming and thermal-assisted forming. Peng et al. [14] investigated the hydraulic bulging process for metallic bipolar plates and found that it yielded a more uniform wall-thickness distribution. However, the smaller the microchannel dimensions, the more challenging the forming process becomes, requiring higher hydraulic pressures. Moreover, many companies have reported that the cycle time for a single hydraulic forming process is relatively long, limiting production efficiency. Although thermal-assisted forming improves material formability, most of the thermal energy is dissipated in heating the die, resulting in extremely low energy utilization.

To address these challenges, novel hybrid forming techniques, such as ultrasonic-vibration, electromagnetic-field, and laser-assisted forming, have garnered significant attention due to their ability to enhance material plastic flow, reduce forming loads, and ultimately improve the quality of sheet-metal forming [15]. Among these, ultrasonic-vibration-assisted microforming typically involves applying vibrations to either the forming die or the material itself, ensuring that the material remains in a vibrational state throughout the plastic deformation process. This technique offers the advantages of high energy efficiency and relatively low equipment costs. In 1955, Blaha and Langenecker [16] observed a “softening phenomenon” in tensile tests on rods subjected to ultrasonic vibration, a phenomenon now known as the Blaha effect. Since ultrasonic vibration induces both volumetric and surface effects in metallic materials, facilitating plastic deformation, it has emerged as a research hotspot in recent years. Since then, a wide range of studies have focused on ultrasonic-vibration-assisted microforming techniques, such as micro-blanking [17,18], micro-deep-drawing [19,20,21], and micro-extrusion [22,23]. However, there is limited research on ultrasonic-vibration-assisted stamping, particularly regarding the effect of ultrasonic vibration and scale coupling on the plastic deformation behavior and buckling characteristics of thin sheets.

This study investigates the effect of thickness and ultrasonic-vibration energy fields on the anti-wrinkling capacity of TA1 titanium sheets using diagonal stretching experiments. Meanwhile, by analyzing the load–displacement curves during the tensile process to observe the forming load and the onset of wrinkling, along with measuring the wrinkle height at buckling locations using a super-depth-of-field microscope, this study explores methods for suppressing compressive deformation instability in TA1 titanium thin sheets. The findings aim to provide valuable insights for process optimization in the manufacturing of ultra-thin bipolar plates.

## 2. Experimental Scheme

### 2.1. Experimental Materials and Specimen Preparation

#### 2.1.1. Experimental Materials

The material used in this experiment was a TA1 titanium thin sheet, manufactured by Baoji Titanium Industry Co., Ltd. (Baoji, China). TA1 titanium foils with thicknesses of 0.075 mm and 0.1 mm were fabricated using a rolling process with an optimized rolling speed and tension control. The mechanical properties of the TA1 titanium foils are listed in Table 1.

#### 2.1.2. Specimen Preparation

**Figure 1:**
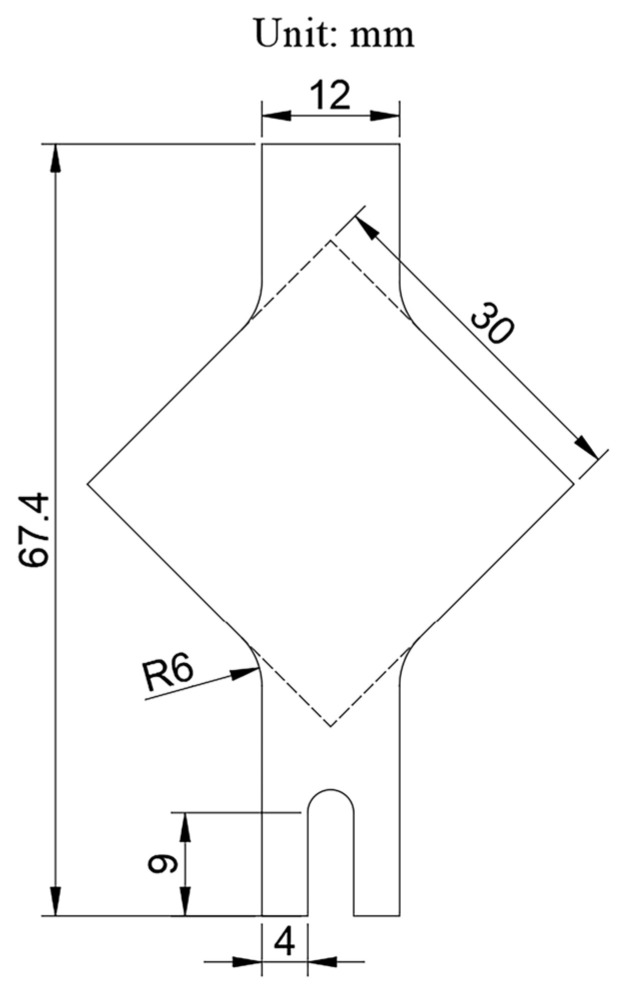
Dimensions of TA1 tensile sample.

**Figure 2:**
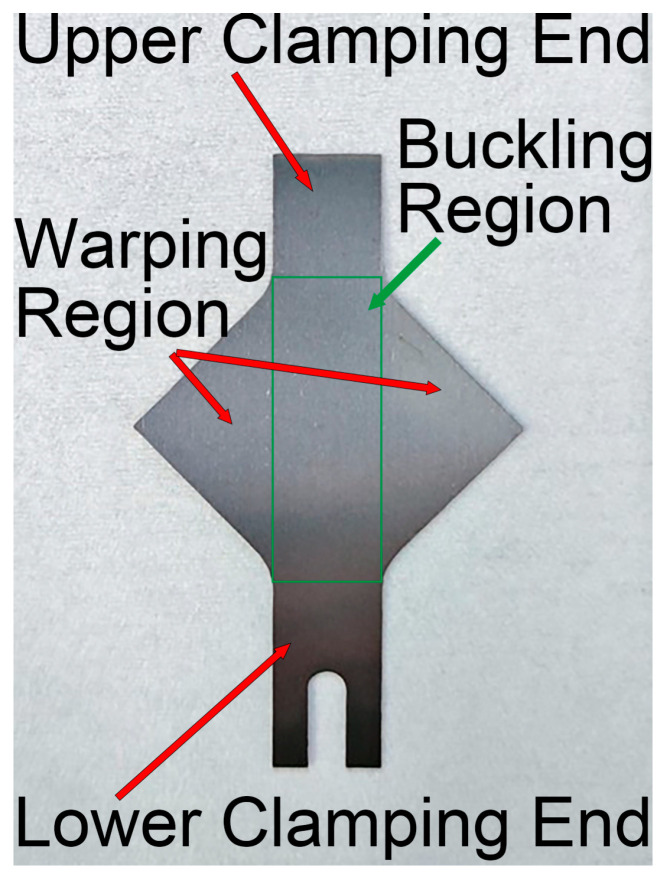
Physical sample.

To accommodate the dimensions of the bipolar plates, the standard YBT sample dimensions were scaled proportionally, and a U-shaped groove was machined at the lower clamping end to facilitate attachment of the ultrasonic transducer. The final dimensions are shown in Figure 1. In the standard YBT sample, buckling occurs within a circular region with a central radius of approximately 30 mm [24]; therefore, the 12 mm × 35.4 mm green rectangular area shown in Figure 2 was selected as the buckling measurement region. The diagonal regions on both sides of the sample, after wrinkling at the buckling area, experienced warping due to uneven loading and were therefore designated as the warping regions.

After determining the dimensions of the tensile specimens, electrical discharge wire cutting (EDM) was employed for machining. Given the extremely thin nature of the TA1 titanium foils used in the experiment, multiple layers of the foils were stacked and clamped between steel plates of appropriate thicknesses to ensure dimensional accuracy and machining efficiency during the cutting process. To achieve a high precision, slow-wire EDM was utilized, with the electrode wire operating at a speed of 0.2 mm/s, a machining current of 5 A, a pulse width of 3 µs, and a pulse interval of 1 µs. Due to the presence of oil residues on the specimens’ surface after wire cutting and the potential for scratches during cutting and transportation, only specimens with smooth, undamaged surfaces were selected for tensile testing. Additionally, to prevent inaccuracies in the experimental results and subsequent analyses, selected specimens were ultrasonically cleaned in a 40 kHz ultrasonic cleaner using alcohol for 1 min to remove contaminants and surface residues.

To evaluate the precision of the machined specimens, five samples of 0.075 mm and 0.1 mm thicknesses were randomly selected for measurement of their thickness and surface roughness. The results, presented in Table 2, Table 3 and Table 4, indicate minimal variation among the specimens, confirming that machining errors are negligible and will not affect the experimental outcomes.

### 2.2. Experimental Apparatus and Methodology

**Figure 3:**
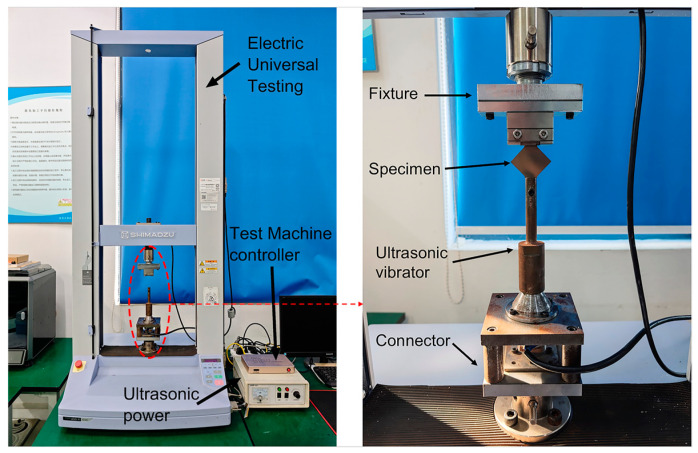
Ultrasonic-vibration-assisted tensile testing device.

The ultrasonic-vibration-assisted uniaxial tensile testing apparatus for titanium thin sheets, as illustrated in Figure 3, primarily consists of a Shimadzu electronic universal testing machine, an ultrasonic-vibration system, and a connecting assembly. The ultrasonic-vibration system comprises ultrasonic power and an ultrasonic vibrator. The power converts conventional AC electricity into high-frequency alternating current, which is then transmitted to the ultrasonic transducer. Utilizing the inverse piezoelectric effect of piezoceramics, the transducer converts these electrical signals into mechanical vibrations of the same frequency.

The upper clamping end of the test specimen was secured using a conventional tensile testing fixture, while the lower end was fastened with a bolt at the front of the ultrasonic vibrator. During the experiment, the testing machine’s displacement was set to 0.8 mm, with a stretching speed of 2 mm/min. Ultrasonic vibrations were applied in the tensile direction under these conditions. The ultrasonic system operated at a fixed frequency of 20 kHz, with sinusoidal vibrations. Amplitude was controlled by adjusting the output current of the power supply, with higher currents producing larger amplitudes. Excessive amplitude could cause rupture at the junction between the clamping end and the deformed region of the specimen. To vary the amplitude, four different current values—0 mA (no vibration), 15 mA, 30 mA, and 45 mA—were used, and each condition was repeated at least three times to ensure consistency and minimize random errors.

### 2.3. Buckling Measurement

**Figure 4:**
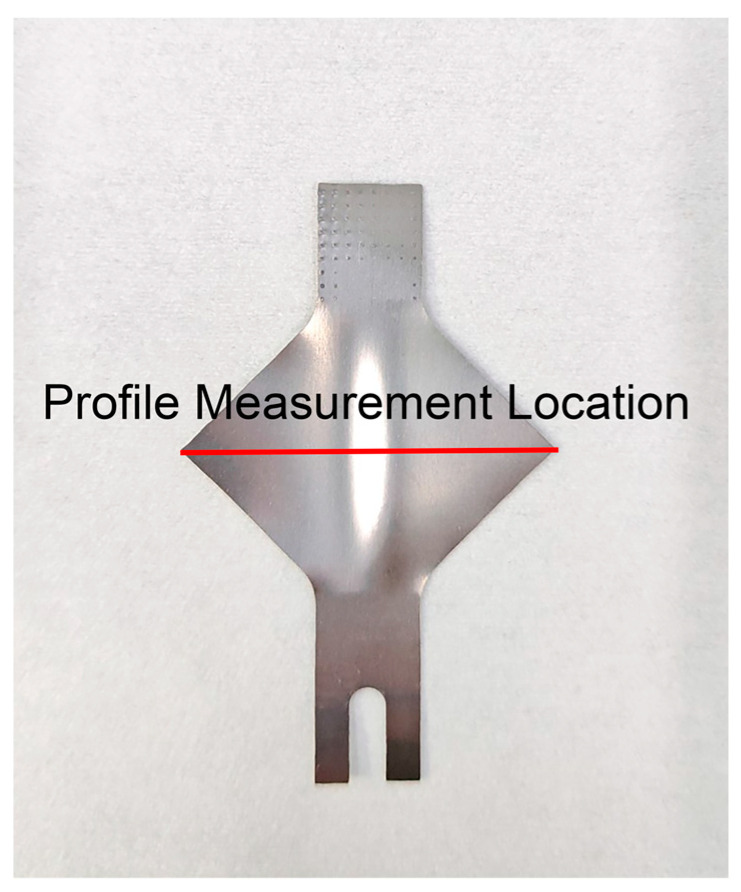
Wrinkled sample.

The surface morphology along the central diagonal of the square plate was measured using a super-depth-of-field microscope for the formed specimens. The corresponding surface profile was obtained, and the wrinkle height variation at the buckling region was recorded. The measurement location along the central diagonal of the square plate is depicted in Figure 4.

## 3. Results and Discussion

To examine the effects of thickness and ultrasonic-vibration energy fields on the anti-wrinkling and forming performance of TA1 titanium sheets, the experimental results are analyzed from two perspectives: the load–displacement curves, and the morphology and height of the wrinkles.

### 3.1. Load–Displacement Curves

**Figure 5:**
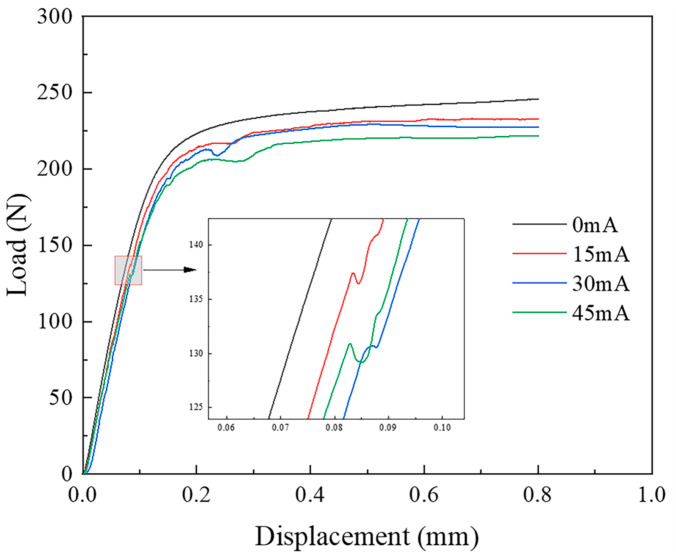
Load–displacement curve of 0.075 mm thick TA1 titanium sheet.

**Figure 6:**
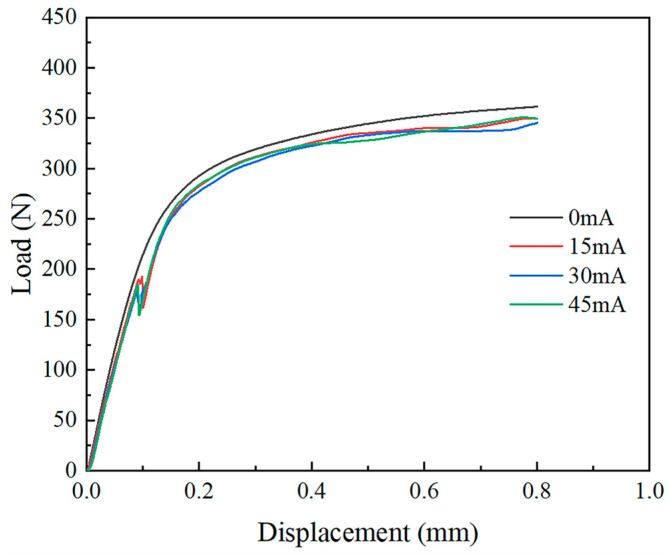
Load–displacement curve of 0.1 mm thick TA1 titanium sheet.

Due to the high vibration frequency in the experiment and the rapid load fluctuations, the limited data acquisition rate of the universal testing machine was insufficient to fully capture the high-frequency vibration data. As a result, irregular anomalies appeared in the load–displacement curve. To enable a more accurate analysis, the anomalous sections of the curve were fitted, and the processed curves are presented in Figure 5 and Figure 6.

In Figure 5 and Figure 6, no inflection points are observed in the load–displacement curves for the two thicknesses of TA1 titanium sheets under static tension. However, following the application of the ultrasonic-vibration energy field, inflection points emerge in both cases. These inflection points correspond to the bifurcation points arising from the transition of the sheet material from in-plane to out-of-plane deformation. According to Hill’s general theory on the uniqueness of elastic–plastic materials [25], bifurcation occurs when the characteristic equation of the variational formulation (Equation (1)) admits non-unique solutions:

(1) $$\delta I=0,I=\int_{V}\left(\Delta_{s_{i\dot{j}}^{*}}+\sigma_{ij}v_{i,j}^{*}\right)v_{i,j}^{*}\mathrm{d}V$$

Therefore, the moment corresponding to the bifurcation point marks the onset of wrinkling in the square sheet. This suggests that the ultrasonic-vibration energy field induces branching buckling. After the bifurcation in the curves, the structure regains its load-bearing capacity as deformation progresses.

A comparison of the load–displacement curves for TA1 titanium sheets of different thicknesses reveals distinct wrinkling behaviors under ultrasonic vibration. For the 0.075 mm thick sheet, an inflection point emerges in the elastic deformation stage at approximately 0.085 mm displacement, whereas for the 0.01 mm thick sheet, the inflection point appears later, at around 0.09 mm displacement. This indicates that as the thickness decreases, the onset of wrinkling occurs earlier, signifying poorer wrinkle resistance. Moreover, both sheet thicknesses exhibit instability within the elastic deformation region after the application of the ultrasonic-vibration energy field. This phenomenon can be attributed to the reduction in both the material’s yield stress and the structural flow stress under ultrasonic excitation. The extent of the reduction in flow stress is influenced by both the vibration frequency and amplitude, with increases in either parameter leading to a more pronounced stress reduction. This reduction is attributed to the acoustic softening and stress superposition effects [15]. Furthermore, a second inflection point appears in the plastic deformation region of the load–displacement curve for the 0.075 mm thick sheet, occurring between the 0.2 mm and 0.3 mm displacement. The degree of load reduction at this point is less severe than at the first inflection point, suggesting that localized secondary wrinkling occurs. This further reinforces the conclusion that the anti-wrinkling performance deteriorates as sheet thickness decreases.

A comparison of the load–displacement curves for 0.075 mm and 0.1 mm thick TA1 titanium sheets under both ultrasonic-vibration-assisted and static stretching conditions demonstrates that ultrasonic vibration significantly reduces the material’s forming load. For the 0.075 mm thick sheet, the forming load decreases progressively with increasing amplitude, reaching a minimum at a current of 45 mA. At this current, the load at a displacement of 0.6 mm is approximately 22 N lower than that observed under the no-vibration condition. For the 0.1 mm thick sheet, the forming load also decreases with ultrasonic vibration, with the minimum forming load occurring at 45 mA. At this current, the load at the 0.6 mm displacement is reduced by about 14 N compared to the no-vibration condition. However, when the current is increased from 15 mA to 30 mA, although the amplitude increases, the load remains largely unchanged. This behavior may be attributed to the relatively larger area and thickness of the specimen, which reduces the effect of the vibration system.

### 3.2. Wrinkle Morphology and Height

**Figure 7:**
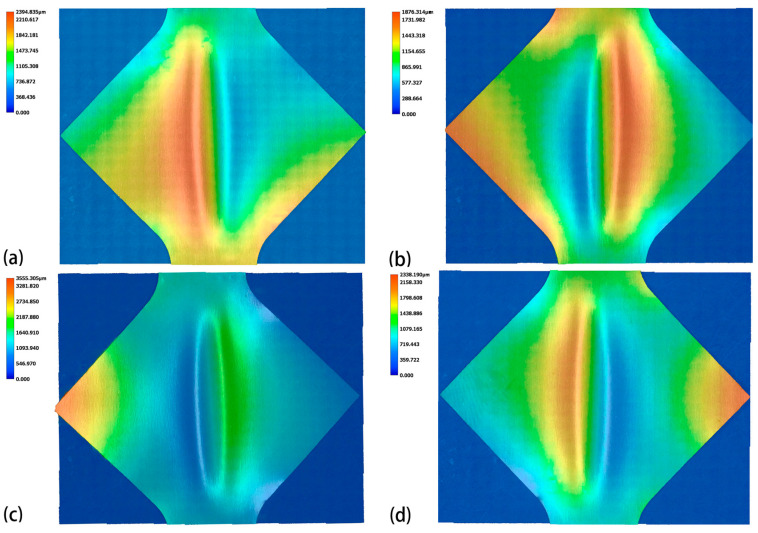
Surface height of 0.075 mm thick TA1 titanium sheet after tensile testing: (**a**) 0 mA, (**b**) 15 mA, (**c**) 30 mA, (**d**) 45 mA.

**Figure 8:**
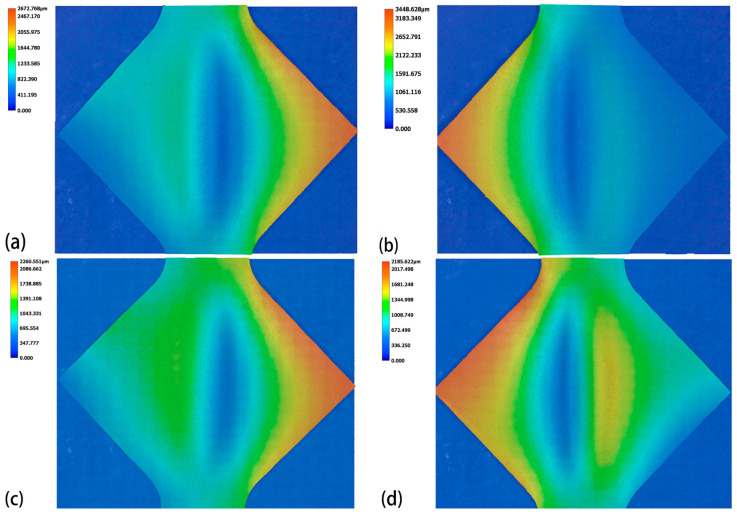
Surface height of 0.1 mm thick TA1 titanium sheet after tensile testing: (**a**) 0 mA, (**b**) 15 mA, (**c**) 30 mA, (**d**) 45 mA.

**Figure 9:**
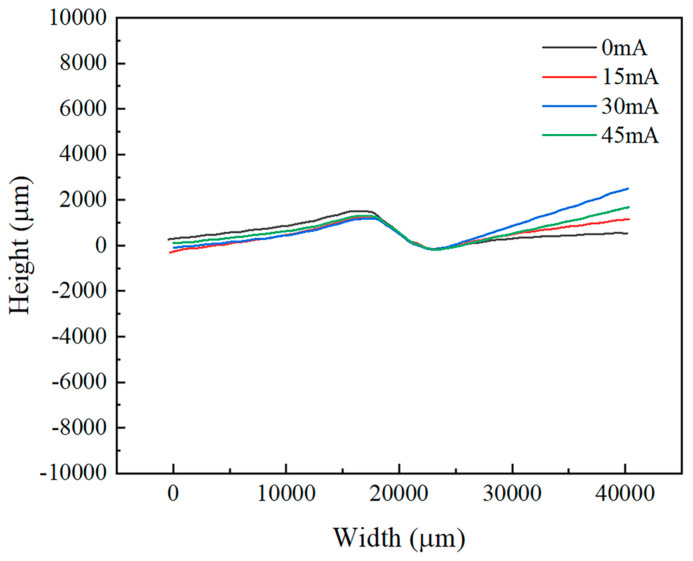
Central profile of 0.075 mm thick TA1 titanium sheet under different amplitudes.

**Figure 10:**
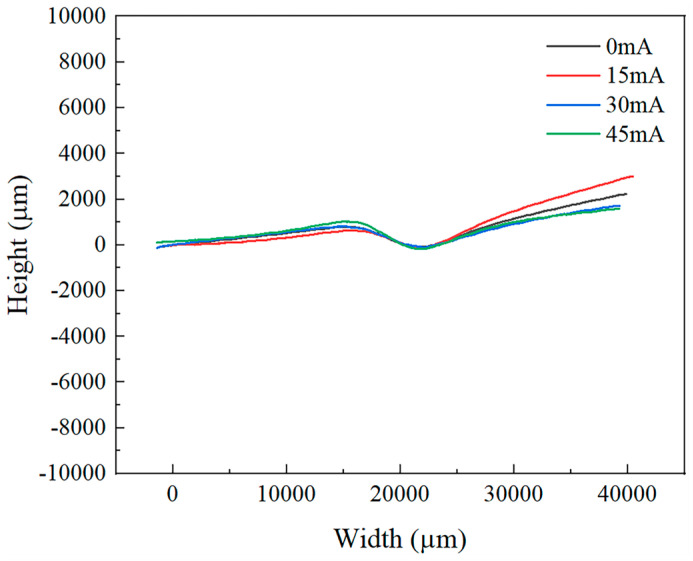
Central profile of 0.1 mm thick TA1 titanium sheet under different amplitudes.

Figure 7 and Figure 8 display the surface height profiles of the two thicknesses of TA1 titanium sheets after stretching, while Figure 9 and Figure 10 illustrates the central profile curve of the wrinkled sheet, measured at the location indicated in Figure 4. From these figures, it is clear that the wrinkle morphology is wave-like, with the buckling region exhibiting a half-wave number of 2, consisting of both a “trough” and a “crest”.

Given the focus on the wrinkling behavior of TA1 titanium sheets, the surface heights at the “crest” and “trough” along the central profile curve were measured. The wrinkle heights at the buckling region for both sheet thicknesses, under varying amplitudes, are depicted in Figure 9 and Figure 10. It is clear that, at the same amplitude, the wrinkle height at the buckling region for the 0.075 mm thick sheet is markedly greater than that for the 0.1 mm thick sheet. In the absence of vibration, the difference in wrinkle height at the buckling region between the two thicknesses reaches 800 μm. This further corroborates the conclusion that wrinkling intensity increases as sheet thickness decreases.

**Figure 11:**
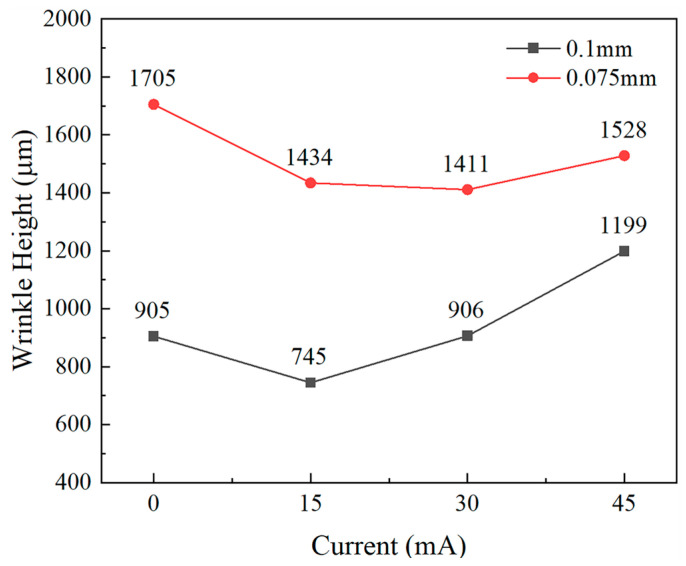
Wrinkle height at the buckling region of TA1 titanium sheets with two thicknesses under different amplitudes.

Figure 11 illustrates the variation in wrinkle height of 0.075 mm and 0.1 mm thick TA1 titanium sheets as the current (and thus the amplitude) increases. Initially, the wrinkle height decreases, but subsequently increases as the current rises. Specifically, for the 0.075 mm thick sheet, the wrinkle height at the buckling region reaches a minimum value of 745 μm at 15 mA. For the 0.1 mm thick sheet, the minimum wrinkle height of 1411 μm occurs at 30 mA. However, at 45 mA, the wrinkle height for the 0.1 mm thick sheet increases by 294 μm. These findings indicate that the ultrasonic-vibration energy field effectively suppresses wrinkling during the titanium sheet-forming process within specific amplitude ranges, thereby providing experimental evidence to support the optimization of bipolar plate processing.

## 4. Conclusions

A comprehensive study has been conducted on the effects of sheet thickness and ultrasonic-vibration energy fields on the anti-wrinkling performance of TA1 titanium sheets. Based on the experimental findings, the following conclusions can be drawn:

1. Analysis of the load–displacement curves and wrinkle height maps for the 0.075 mm and 0.1 mm thick TA1 titanium sheets reveals that thinner sheets exhibit inferior anti-wrinkling performance and may experience localized secondary wrinkling.
2. The ultrasonic-vibration energy field can induce branching buckling in the diagonal stretching test of square plates, allowing the onset of wrinkling in TA1 titanium sheets to be determined from the inflection points in the load–displacement curves.
3. The ultrasonic-vibration energy field reduces both the yield and flow stresses of the material, facilitating wrinkle formation during the elastic deformation phase. As the amplitude increases, the stress reduction becomes more pronounced.
4. At the same vibration frequency, the wrinkle height of the TA1 titanium sheets initially decreases and then increases as the amplitude increases. Specifically, the 0.075 mm thick sheet reaches its minimum wrinkle height at 15 mA, while the 0.1 mm thick sheet does so at 30 mA. Moderate ultrasonic amplitudes effectively suppress wrinkling, while excessively high amplitudes may exacerbate it.
5. The ultrasonic-vibration energy field reduces the forming load of TA1 titanium sheets and, within an optimal amplitude range, enhances both the forming quality and anti-wrinkling performance. Therefore, the careful selection of ultrasonic amplitude and sheet thickness parameters can optimize ultrasonic-vibration-assisted stamping, thereby improving the forming quality and engineering applicability of bipolar plates for hydrogen fuel cells.

## Figures and Tables

**Table 1:** Mechanical properties of TA1 titanium material.

Thickness (mm)	Elastic Modulus (Gpa)	Yield Strength (Mpa)	Tensile Strength (Mpa)	Elongation After Fracture (%)	Hardening Index n
0.075	82.6	231.3	291.4	44	0.11
0.1	65.7	229.6	300.2	36	0.13

**Table 2:** The thickness measurement results of the 0.075 mm thick specimens.

Measurement Location	Measured Values (mm)	Mean Value (mm)	Variance (mm^2^)
Buckling Region	0.074 0.073 0.074 0.075 0.072	0.0736	0.00000104
Warping Region	0.075 0.074 0.075 0.073 0.075	0.0744	0.00000064

**Table 3:** The thickness measurement results of the 0.1 mm thick specimens.

Measurement Location	Measured Values (mm)	Mean Value (mm)	Variance (mm^2^)
Buckling Region	0.100 0.099 0.099 0.098 0.097	0.0986	0.00000104
Warping Region	0.098 0.098 0.099 0.098 0.099	0.0984	0.000000168

**Table 4:** Surface roughness (Ra) measurement results.

Specimen Thickness (mm)	Measured Values (µm)	Mean Value (µm)	Variance (µm^2^)
0.075	0.074 0.062 0.056 0.070 0.075	0.0674	0.00005374
0.1	0.051 0.042 0.055 0.057 0.052	0.0474	0.00008584

## Data Availability

The original contributions presented in the study are included in the article, further inquiries can be directed to the corresponding authors.
